# Chemopreventive effects and anti-tumorigenic mechanisms of *Actinidia arguta*, known as sarunashi in Japan toward 4-(methylnitrosamino)-1-(3-pyridyl)-1-butanone (NNK)- induced lung tumorigenesis in a/J mouse

**DOI:** 10.1186/s41021-022-00255-0

**Published:** 2022-12-09

**Authors:** Jun Takata, Naoko Miyake, Yusuke Saiki, Misako Tada, Kensuke Sasaki, Toshio Kubo, Katsuyuki Kiura, Sakae Arimoto-Kobayashi

**Affiliations:** 1 Faculty of Medicine, Dentistry and Pharmaceutical Sciences, Okayama University, Okayama, 700-8530 Japan; 2 Faculty of Pharmaceutical Sciences, Okayama University, Okayama, 700-8530 Japan; 3 Department of Allergy and Respiratory Medicine, Okayama University Hospital, Okayama, 700-8530 Japan

**Keywords:** *Akt* signal transduction, Lung tumorigenesis, Anti-mutagenesis, DNA methylation, Tobacco-specific nitrosamine, Isoquercetin

## Abstract

**Background:**

Previously, we reported the inhibitory effect of *Actinidia arguta* juice, known as sarunashi juice (sar-j) in Japan, on mutagenesis, inflammation, and mouse skin tumorigenesis. The components of *A. arguta* responsible for the anti-mutagenic effects were identified to be water-soluble, heat-labile phenolic compounds. We proposed isoquercetin (isoQ) as a candidate anticarcinogenic component. In this study, we sought to investigate the chemopreventive effects of *A. arguta* juice and isoQ on 4-(methylnitrosamino)-1-(3-pyridyl)-1-butanone (NNK)-induced lung tumorigenesis in A/J mice, and identify the possible mechanisms underlying the anti-tumorigenic effects of *A. arguta*.

**Results:**

The number of tumor nodules per mouse lung in the group injected with NNK and administered *A. arguta* juice orally was significantly lower than that in the group injected with NNK only. Oral administration of isoQ also reduced the number of nodules in the mouse lungs. As expected, the mutagenicity of NNK and 1-methyl-3-nitro-1-nitrosoguanidine (MNNG) detected using *S. typhimurium* TA1535 decreased in the presence of sar-j. However, NNK and MNNG mutagenicity detected using *S. typhimurium* YG7108, a strain lacking the O^6^-methylguanine DNA methyltransferases (*ogt*_*ST*_ and *ada*_*ST*_) did not decrease in the presence of sar-j suggesting that sar-j may mediate its antimutagenic effect by enhancing the DNA damage repair by *ogt*_*ST*_ and *ada*_*ST*_. Phosphorylation of Akt, with or without epidermal growth factor stimulation, in A549 cells was significantly decreased following sar-j and isoQ treatment, indicating that components in sar-j including isoQ suppressed the PI3K/AKT signaling pathways.

**Conclusions:**

Sar-j and isoQ reduced NNK-induced lung tumorigenesis. Sar-j targets both the initiation and growth/progression steps during carcinogenesis, specifically via anti-mutagenesis, stimulation of alkyl DNA adduct repair, and suppression of Akt-mediated growth signaling. IsoQ might contribute in part to the biological effects of sar-j via suppression of Akt phosphorylation, but it may not be the main active ingredient.

**Supplementary Information:**

The online version contains supplementary material available at 10.1186/s41021-022-00255-0.

## Introduction

Dietary factors can considerably influence cancer risk in humans. Epidemiological data support the association between high fruit intake and a low risk of chronic diseases [1]. The bioactive properties of fruits have long been the focus of investigations. For example, grapes (*Vitis* spp.), pomegranate (*P. granatum*), and blueberries (*Vaccinium* spp.) have been reported as good dietary sources of bioactive compounds with health benefits against chronic diseases [2–4]. Previously, we reported the anticarcinogenic effects of the juice of *Vitis coignetiae*, crimson glory vine, a wild grape known as yamabudo, in Japan [5, 6]. We also reported the inhibitory effect of *Actinidia arguta* juice, known as sarunashi in Japan, on mutagenesis, inflammation, and mouse skin tumorigenesis [7]. The anti-mutagenic and anticarcinogenic mechanisms, and anti-oxidant activity of *A. arguta* juice was determined using the free radical scavenging assay. *A. arguta* juice was found to inhibit the metabolic activation of mutagens with phase I enzymes, and possess anti-inflammatory activity [7]. *A. arguta* juice conferred neuroprotection to delay or prevent neurodegeneration in Parkinson’s disease [8]. *A. arguta* is reported to be one of the richest sources of polyphenols and vitamin C. A pharmaceutical composition of *A. arguta*, *A. kolomikta*, and *A. polygama* extracts has already been registered for the prevention and treatment of some immune (inflammatory) mediated diseases, as well as for the treatment of some non-allergic inflammatory diseases [9]. The major polyphenolic components of *A. arguta* were identified to be isoquercetin (hereafter referred to as isoQ) and hyperoside, which are present at 1.99 g and 2.17 g per 100 g of the polyphenolic fraction of *A. arguta*, respectively [10]. Orfali reviewed the potential anticancer mechanisms of isoQ and concluded that the underlying mechanism involved interactions between several signaling pathways [11]. We performed partial purification of the antimutagenic components in *A. arguta*, and found that the components responsible for the antimutagenic effects were water-soluble and heat-labile phenolic compounds [7]. Although the exact identity of the components responsible for antimutagenic and anticarcinogenic properties in *A. arguta* remain unclear, we hypothesized that isoQ is a candidate anticarcinogenic component.

Lung cancer accounts for a major proportion of cancers [12]. The relationship between lung cancer and tobacco-specific nitrosamine, 4-(methylnitrosamino)-1-(3-pyridyl)-1-butanone (NNK), has been investigated via molecular epidemiological studies [13]. NNK effectively induces lung tumors in mice, rats, and hamsters [14] and is believed to play a significant role in the development of lung cancer in smokers.

The phosphoinositide-3-kinase (PI3K)/AKT pathway has a decisive role in cancerous cells development and survivability, and Akt locate the downstream of the signal pathway from EGF via PI3K. Barron et al. reported that red wine significantly reduced basal and EGF-stimulated Akt and Erk phosphorylation in the human lung epithelial-like cell line A549 cells [15]. A bioactive flavonoid, baicalein significantly suppressed the PI3K/Akt/NF-κB pathway in A549 cells [16]. A anthraquinone, purpurin eliminates the A549 lung cancer cells by blocking the PI3K/AKT pathway [17]. Park and Seol reported pretreatment of A549 cells to EGF-R inhibitor down-regulated the protein levels of total Akt and phosphorylated active Akt [18]. Sar-j and isoQ may affect the PI3K/AKT signaling pathways, and EGF-simulation may influence the effects of sar-j and isoQ on the pathway. We investigated the effects of sar-j and isoQ on PI3K/AKT signaling pathways with or without EGF stimulation.

In this study, we investigated the chemopreventive effects of *A. arguta* juice and isoQ on NNK-induced lung tumorigenesis in A/J mice. Furthermore, to identify the mechanisms underlying the anti-tumorigenic effects of *A. arguta* and isoQ, we investigated their antimutagenic effects on the alkylating agents NNK and 1-methyl-3-nitro-1-nitrosoguanidine (MNNG), and effects on DNA-alkylation, DNA repair with O^6^-methylguanine DNA methyltransferases, and on Akt-mediated the growth signaling.

## Materials and methods

### Materials

*A. arguta* (sarunashi) fruits were purchased from local stores in Shin-jo village in Okayama prefecture (Japan). The average weight of a sarunashi fruit was 7.27 ± 1.72 g (mean ± standard deviation). The fruits were processed using a juicer, centrifuged at 2600×g for 20 min at 20 °C, and the supernatant was collected. Sarunashi fruits (200 g) yielded 65.5 g (63 mL) of supernatant (hereafter referred to as sarunashi juice or sar-j). Sar-j was stored at − 20 °C until use. IsoQ (CAS 21637–25-2, MW 464.38) was purchased from Kanto Chemical Co. Inc. (Tokyo, Japan). NNK was purchased from Toronto Research Chemicals (North York, ON, Canada) and MNNG was purchased from Nacalai Tesque (Kyoto, Japan). Vitamin C was purchased from Wako Pure Chemical Co. Ltd. (Osaka, Japan). 3-(4,5-Dimethyl-2-thiazolyl)-2,5-diphenyl-2H-tetrazolium bromide (MTT) was purchased from Dojindo Laboratories (Kumamoto, Japan). For metabolic activation, the supernatant fraction of rat liver homogenate (S9) prepared using phenobarbital and 5,6-benzoflavone was obtained from FUJIFILM Wako Pure Chemical. Akt (pan) (C67E7) rabbit mAb (#4691), phospho-Akt (Ser473) (D9E) rabbit mAb (#4060), and LY294002 (#9901) were purchased from CST, Japan (Tokyo, Japan). *Salmonella enterica subspecies I, serovar Typhimurium* (*Salmonella typhimurium*) strain TA1535 [*hisG46 ΔuvrB gal bio chl1005 rfa1001*], was a gift from Dr. B. N. Ames of the University of California, Berkeley [19]. *S. typhimurium* YG7108 [*hisG46 ΔuvrB gal bio chl1005 rfa1001 Δada*_*st*_*::Km*^*r*^ *Δogt*_*st*_*::Cm*^*r*^], a strain lacking O^6^-methylguanine DNA methyltransferases (*ogt*_*ST*_ and *ada*_*ST*_), was a kind gift from Dr. M. Yamada of the National Institute of Health [20]. The human lung epithelial-like cell line A549 (ATCC CCL185), derived from lung carcinoma, was provided by RIKEN BRC through the National BioResource Project of the MEXT/AMED, Japan (Tsukuba, Japan).

The vitamin C content in sar-j was quantitatively determined by iodine titration [21] and average vitamin C content in sar-j samples was found to be 1.48 ± 0.030 g/L. The total amount of polyphenolics in the juice was measured using the method of Singleton and Rossi [22] using the Folin-Ciocalteu method in terms of the gallic acid equivalent (mg/mL), and the average total phenolics amount in sar-j was found to be 4.40 ± 0.041 mg/mL.

### Animals

Mice (A/J jms Slc female, 3-weeks old, average weight 8–13 g) were purchased from Japan SLC Inc. (Hamamatsu, Japan). Five mice were housed per cage in the animal room and randomly separated into treatment groups at least 1 week prior to the commencement of experiments. The mice had free access to murine chow pellets (MF powder, Oriental Yeast Co. Ltd., Tokyo, Japan) and water, and were maintained on a 12-h light/12-h dark cycle with optimum air exchange and a constant room temperature of 20 °C. All experiments were performed in accordance with the Guidelines for Animal Experiments of the Okayama University Advanced Science Research Center (permission no. OKU-2018028, 2,018,030, 2,019,671, 2,021,460, 2,021,461, 2,022,299, and 2,022,336) based on the Act on Welfare and Management of Animals (Act of Japan, No. 105 of October 1, 1973, and Amendment of Act No. 68 of 2005), Standards Relating to the Care, Management, and Alleviation of Pain and Distress of Laboratory Animals (Notice of the Ministry of the Environment No. 88 of 2006).

### Anti-tumorigenesis study in mice with Sar-j and isoQ

NNK-induced tumorigenesis experiments were performed as described in our previous report [5]. In experiment 1, mice (A/J, 4-weeks old) were divided into three groups of 15–16 animals each (groups I–III). Prior to the experiments, sar-j was defrosted, centrifuged at 9000×g for 20 min at 20 °C, and the supernatant was collected. Potassium disulfite (0.1 g) was added to 1 L of the supernatant to prevent fermentation. Mice in group I received water containing potassium disulfite (0.1 g/L). Mice in groups II and III received sar-j in lieu of water from 4 weeks of age to the time of sacrifice. Tumors were induced in groups I and II with a single intraperitoneal (i.p.) injection of NNK (100 mM in 0.1 ml saline) at 8 weeks of age. The mice in group III were injected with 0.1 mL of saline as a substitute for NNK (control). The mice were sacrificed at 30 weeks of age.

In experiment 2, two isoQ doses of 25 mM (11.6 mg/L) and 10 mM (4.64 mg/L) were tested. Because the average total phenolics content in sar-j was 4.40 ± 0.041 g/L, we selected approximately 0.5 and 0.1% of the total phenolics content in sar-j. Mice (A/J, 4-weeks old) were divided into five groups of 5–15 animals each (groups IV–VIII). At 8 weeks of age, mice in groups IV, V and VI were injected with NNK (100 mM in 0.1 ml saline), while the mice in groups VII and VIII were administered a single i.p. injection of 0.1 mL saline. The mice received 10 mM isoQ (groups V) and 25 mM of isoQ (group VI and VII) in lieu of water from 4 weeks of age until sacrifice. The mice were sacrificed at 30 weeks of age. In both experiments, the number of surface lung nodules of the left lung lobe was counted using a loupe and digital calipers (AS ONE Corporation, Osaka, Japan). Tumor nodules that were > 1 mm in diameter were counted.

### Anti-mutagenicity test

The inhibitory effects of sar-j and isoQ on NNK- and MNNG-induced mutagenicity were investigated using the Ames test [19]. Sar-j was defrosted, centrifuged at 9000×g for 20 min at 20 °C, sterilized by filtration of the supernatant through a 0.45 mM filter, then used for the Ames assay. NNK was assayed with *S. typhimurium* TA1535 or YG7108 in the presence of rat liver homogenate (hereafter refer to as +S9). MNNG was assayed with *S. typhimurium* TA1535 or YG7108 in the absence of rat liver homogenate (hereafter refer to as -S9). The effects of sar-j and isoQ on mutagenicity were examined as previously described [23]. Briefly, the preincubation mixture was prepared by mixing the components in the following order: 100 μL of a sample (sar-j with water, or isoQ solution), 450 μL of S9 mix or Na-phosphate buffer (0.1 M, pH 7.40), 100 μL of an overnight culture of bacteria, and finally 50 μL of a solution of mutagen (NNK or MNNG). Following incubation for 20 min at 37 °C, molten agar was added and the mixture was poured onto a minimal agar plate. The plates were incubated for 48 h, and the resulting revertant colonies were counted manually. When the number of colonies per plate exceeded 3000, the colonies in a specific square area were counted, and the total number of colonies in the plate was estimated from the average count in five such areas. The sample amounts used for the assay are referred to as mL eq. of the original juice. The results of the pilot Ames tests revealed that ≥20 mL of sar-j was cytotoxic on the Ames bacteria (*S. typhimurium*). Therefore, the anti-mutagenicity tests were performed with ≤10 mL of sar-j. Because the total phenolics content in 10 mL of sar-j was 44 mg, up to 116 mg (250 nmol) of isoQ was used for the antimutagenicity test. The experiments were performed in triplicate.

The mutagenic activity (%) was derived using the following equation:

$$100\times \left[\left(\textrm{revertants}\ \textrm{in}\ \textrm{the}\ \textrm{presence}\ \textrm{of}\ \textrm{juice}\ \textrm{or}\ \textrm{isoQ}\right)-\left(\textrm{spontaneous}\ \textrm{revertants}\right)\right]/\left[\left(\textrm{revertants}\ \textrm{in}\ \textrm{the}\ \textrm{absence}\ \textrm{of}\ \textrm{juice}\ \textrm{or}\ \textrm{isoQ}\right)-\left(\textrm{spontaneous}\ \textrm{revertants}\right)\right]$$

### Effects on phosphorylation of Akt in A549 cells

To determine the concentration of sar-j and isoQ for phosphorylation studies, cell viability was measured using the MTT assay as previously described [5]. To evaluate the cytotoxic effects of sar-j and isoQ, A549 cells (1 × 10^4^ cells in 90 μL) were seeded in 96-well plates. Sar-j was diluted to obtain various concentrations ranging from 1 to 10%, of the original juice, hereafter referred to as 0.01 to 0.1 equivalent (eq.), respectively. After 24 h of culture, various concentrations of sar-j or isoQ (10 μL/ well) were added and the cells were incubated for another 24 h. MTT solution (0.05 g in 10 μL) was added to each well and incubated for 2 h. MTT containing medium was then removed and dimethyl sulfoxide (DMSO) was added to the wells to dissolve the formazan complexes. The optical density of the cells were measured and that of the control (untreated) cells was set at 100% viability.

Akt phosphorylation in A549 cells was determined as follows [5]. Briefly, A549 cells were cultured in 35 mm dishes for 24 h and then 0.3 mL of isoQ (final conc., 0.1 mM) or sar-j (final conc., 0.05 equivalent of original juice) dissolved in DMEM were added, and the cells were incubated for 24 h. For the negative control (NC), A549 cells were cultured and 0.3 mL DMEM (without sar-j or isoQ) was added to the cells. For the positive control (PC), A549 cells were cultured and positive kinase-inhibitor control (without sar-j or isoQ) was added to the cells. LY294002 (final 50 mM) served as positive kinase-inhibitor control was added to the PC dish 1 h prior to termination of incubation. For the epidermal growth factor (EGF)-stimulation experiments, EGF (final concentration, 100 ng/mL) was added 10 min before the termination of incubation. Following incubation, the cells were harvested, lysed, and protein concentrations were quantified using a DC Protein Assay kit (Bio-Rad, Hercules, CA, USA). For western blotting, equal amounts of protein samples were electrophoretically separated on a sodium-dodecyl sulfate-polyacrylamide (12%) gel and transferred onto a polyvinylidene difluoride membrane. The membranes were blocked with 5% skim milk in tris buffered saline containing 0.1% Tween 20 (TBST) for 1 h, and then probed with specific antibodies at 4 °C for 16 h. After washing with TBST, the membranes were incubated with appropriate HRP-conjugated secondary antibodies (1:10000) for 1 h at room temperature. Protein bands were detected using ImmunoStar® LD (Fujifilm Wako Pure Chemical, Osaka, Japan) chemiluminescent reagent and imaged with the biomolecular imager ImageQuant LAS 4000 (GE Healthcare Bio-Sciences AB, Uppsala, Sweden). β-Actin was used as a loading control.

### Statistical analyses

Data are expressed as mean ± standard deviation (SD) for each data point as indicated in each fig. SD is indicated with a bar. Statistical significance was set at *P* < 0.05. Statistical analyses were performed using KaleidaGraph (Synergy Software, Reading, PA, USA) and Excel add-in software, Excel statistics (SSRI Co. Ltd., Tokyo, Japan).

## Results

### Anti-tumorigenesis study with Sar-j and isoQ

In experiments 1 and 2, no significant differences in animal growth or noticeable clinical signs of illness were observed among mice throughout the anti-tumorigenesis study (data not shown). No significant difference in food intake or weight gain was observed among group I-VIII. The average amount of water consumed was approximately 20 mL/week/mouse. No significant difference in water consumption, including intake of water, sar-j or isoQ, was observed among group I–VIII.

In experiment 1, the NNK-injected mice in group I (PC) developed a significant number of nodules on the surface of the lungs (5.14 ± 1.46, significantly different from Group VIII, *P* < 0.05) (Table 1). The number of nodules per mouse (0.733 ± 0.799) in group II, which was administered NNK and sar-j, was significantly decreased compared to that in group I (Table 1, *p* < 0.005). Seven out of the 15 mice in group II had no nodules on the lung surface. The average size of the nodules in group II was not significantly smaller compared to that in group I. No mice in group III developed lung nodules following oral administration of sar-j.

**Table 1:** Incidence and average size of lung nodules of the left lung lobe in mice

Group	Treatment	n	Average body weight (g) at 30 wks	% of mice with nodules	No. of nodules per mouse	Average size of nodules (mm)
Exp. 1						
I	NNK *ip* + water	16	25.2 ± 2.2	100	5.14 ± 1.46	1.28 ± 0.22
II	NNK *ip* + sar-j	15	20.3 ± 0.94	53.3	0.733 ± 0.799*	1.18 ± 0.13
III	saline *ip* + sar-j	15	22.4 ± 2.4	0	0 ± 0	0 ± 0
Exp.2						
IV	NNK *ip* + water	15	29.5 ± 3.48	100	3.67 ± 2.23	1.43 ± 0.382
V	NNK *ip* + isoQ 10 mM^1^	10	31.5 ± 3.84	100	3.50 ± 1.08	1.43 ± 0.274
VI	NNK *ip* + isoQ 25 mM^1^	10	32.2 ± 2.20	90	2.00 ± 1.41^#^	1.42 ± 0.328
VII	saline *ip* + isoQ 25 mM^1^	5	35.9 ± 3.40	0	0	0
VIII	saline *ip* + water	10	32.7 ± 3.64	0	0	0

^1^: isoQ 10 mM (4.64 mg/L), 25 mM (11.6 mg/L)

*: *P* < 0.005, significantly different from Group I

^#:^ *P* < 0.05, significantly different from Group IV

In experiment 2, the NNK-injected mice in group IV (PC) developed a significant number of nodules on the surface of the lungs (3.67 ± 2.23, significantly different from Group VIII, *P* < 0.05) (Table 1). The number of nodules per mouse (2.00 ± 1.41) in group VI, which was administered NNK and isoQ (25 mM) was significantly decreased compared with that in group IV (Table 1, *p* < 0.05). The average size of nodules in groups V and VI was not significantly smaller compared to that in group IV. None of the mice in group VII developed lung nodules following oral administration of isoQ. No lung nodules were observed in the mice in group VIII without NNK, sar-j, or isoQ.

**Fig. 1:**
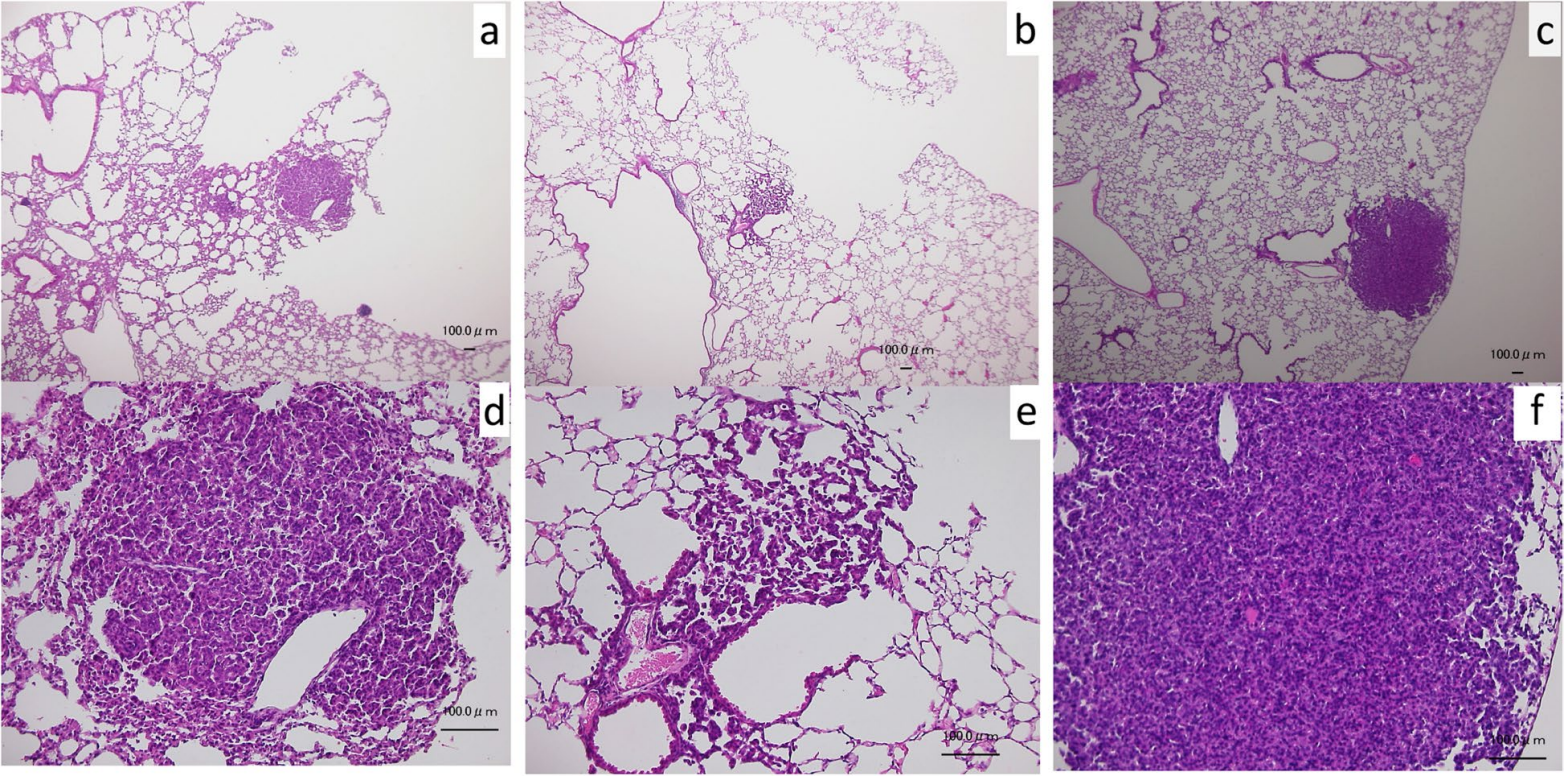
A representative tumor (adenoma/adenocarcinoma) corresponding to the nodule counted macroscopically and the alveolar area around the tumor in the A/J mouse at 30 weeks of age treated with NNK alone (group I) (Fig.1a). NNK + sar-j (group II) (Fig. 1b), or NNK + isoQ (group VI) (Fig. 1c) stained by Hematoxylin and Eosin. Figure 1d, e and f stand for the high magnification of the tumor in Fig. 1d, e and f, respectively. Bar. 100 μm

**Fig. 2:**
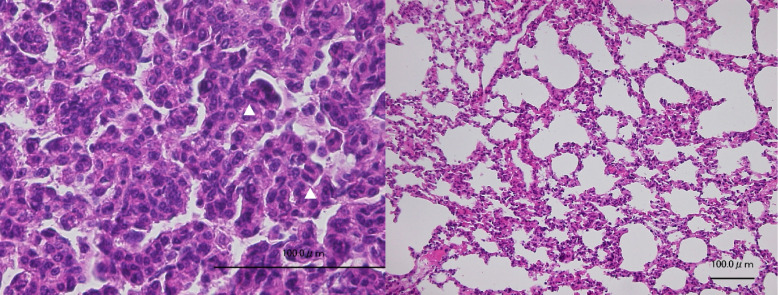
NNK-induced pulmonary lesion (group I) in the A/J mouse at 30 weeks of age. Figure 2a, higher magnification of the tumor in Fig. 1a and 1d. Tumor cells with prominent nuclei with mitotic figures (white head) are observed. Figure 2b, high magnification of alveolar area from another portion from the same slide, a wide range of alveolar area shows hyperplasia/atypical adenomatous hyperplasia. Bar. 100 μm

This NNK-induced tumorigenesis model is a substantial system to detect the chemopreventive effect on lung tumorigenesis by NNK [24, 25]. Histology of the lungs in the A/J mouse at 30 weeks of age treated with NNK alone (group I), NNK + sar-j (group II) or NNK + isoQ (group VI) was investigated (Figs. 1 and 2). Tumors in each group were found. The number of the tumors in the slide confirmed by H&E staining (group II) was significantly few (mean+/−SD, 0.67+/− 0.87/one slide from one mouse, *n* = 9 slides. No tumor slide, *n* = 5/9 slides) compared with that of group I (2.44+/− 0.73/one slide from one mouse, n = 9 slides. No tumor slide, *n* = 0/9 slides) and group IV. (2.4+/− 0.55/one slide from one mouse, n = 5 slides No tumor slide, n = 0/5 slides). Of note, sar-j reduced NNK induced pulmonary nodules by 25,4% on average and completely inhibited the tumor formation in 5 out of 9 mice. As shown in Figs. 1a, b, c, and 2b, alveolar hyperplasia/atypical adenomatous hyperplasia was extremely limited (group II) when compared to the groups I and VI (Fig. 1). Grade of malignancy in the tumor cells (group II) was low when compared to the groups I (Fig. 1).

### Anti-mutagenesis study

Alkylating agents, including NNK, initiate their actions at the DNA level by forming alkyl adducts of DNA, such as, O^6^-alkylguanine DNA adducts. If these adducts are not removed, they mispair with the wrong base during DNA replication, resulting in a mutation. We investigated whether sar-j and isoQ inhibit the mutagenicity of the alkylating agents, NNK and MNNG.

**Fig. 3:**
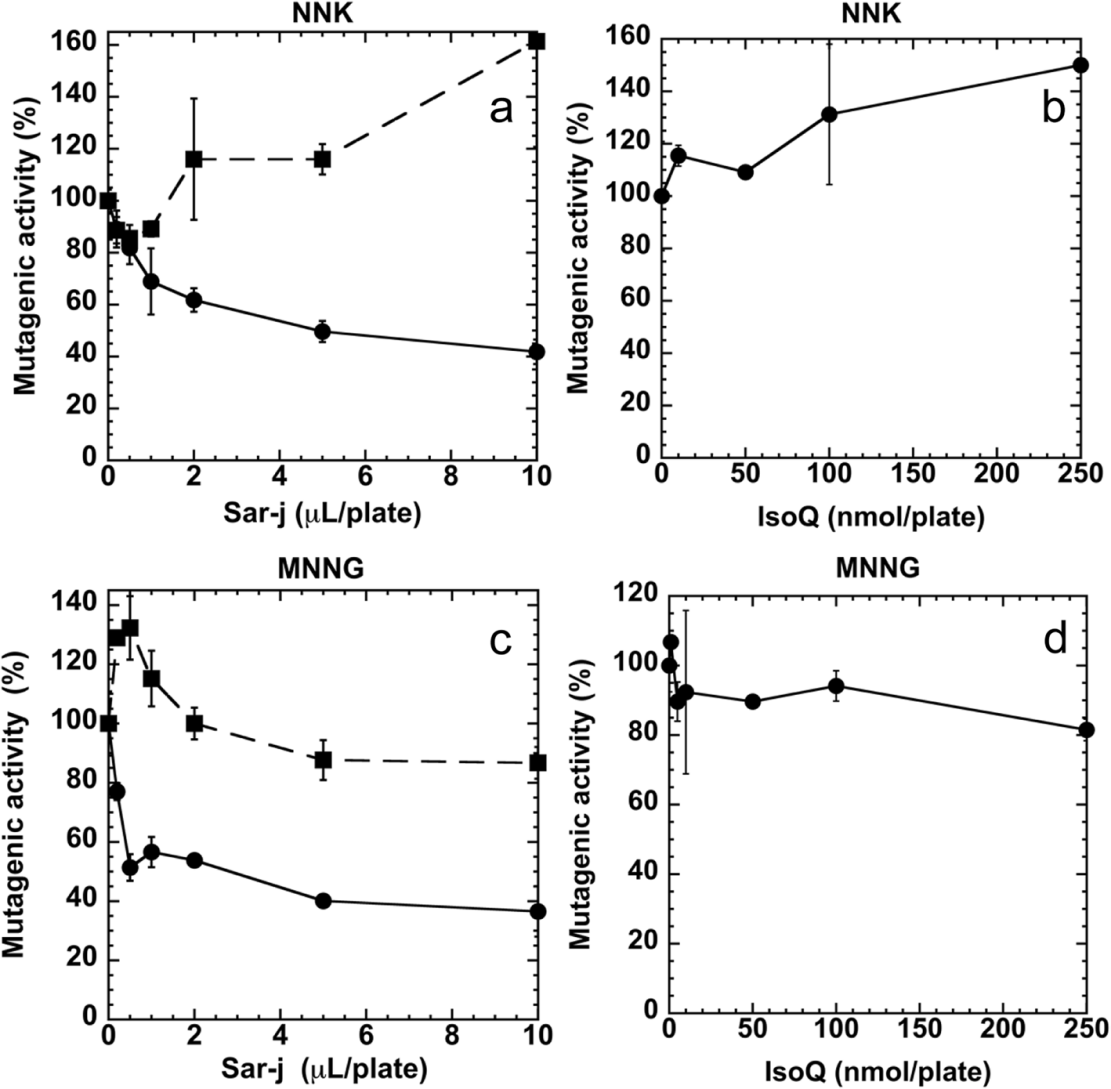
Effect of sar-j (**a**, **c**) and isoQ (**b**, **d**) on the mutagenicity of 4-(methylnitrosamino)-1-(3-pyridyl)-1-butanone (NNK) (**a**, **b**) and 1-methyl-3-nitro-1-nitrosoguanidine (MNNG) (**c**, **d**), respectively. Anti-mutagenicity was assayed with the Ames test using *S. typhimurium* TA1535 (circle) and YG7108 (square, broken line). Experiment was repeated twice and SD is indicated with bar (*n* = 3)

The number of His^+^ revertants/plate in the absence of sar-j or isoQ was found to be 303 ± 10 for 2 μmol of NNK (*S. typhimurium* TA1535,+S9), 846 ± 6.1 for 100 nmol of NNK (*S. typhimurium* YG7108, +S9), 9632 ± 373 for 4 nmol of MNNG (*S. typhimurium* TA1535, −S9), and 2049 ± 204 for 0.25 nmol of MNNG (*S. typhimurium* YG7108, −S9). The number of spontaneous revertants/plate (NC) for *S. typhimurium* TA1535 was 7.5 ± 2.7 (+S9) and 2.5 ± 0.7 (−S9), and that for *S. typhimurium* YG7108 was 13.5 ± 2.1 (+S9) and 12.0 ± 2.8 (−S9). Sar-j and isoQ did not show mutagenic activity toward *S. typhimurium* TA1535 or YG7108 with or without metabolic activation (data not shown). NNK mutagenicity detected using TA1535 was decreased in the presence of sar-j (Fig. 3a, circle), but was not inhibited in the presence of isoQ (Fig. 3b). The amount sar-j required for 50% inhibition (ID_50_) of NNK mutagenicity detected with TA1535 was approximately 5 μL/plate (Fig. 3a, circle). Sar-j also inhibited the mutagenicity of MNNG detected using TA1535 (Fig. 3c, circle), and the amount of sar-j required for 50% inhibition (ID_50_) of MNNG mutagenicity detected with TA1535 was approximately 0.5 μL/plate. However, isoQ did not inhibit MNNG mutagenicity (Fig. 3d). In contrast to the results obtained using TA1535, NNK (Fig. 3a, square), and MNNG (Fig. 3c, square) mutagenicity detected with YG7108 did not decrease in the presence of sar-j.

### Effects of Sar-j and isoQ on cell viability and phosphorylation of Akt in A549 cells

**Fig. 4:**
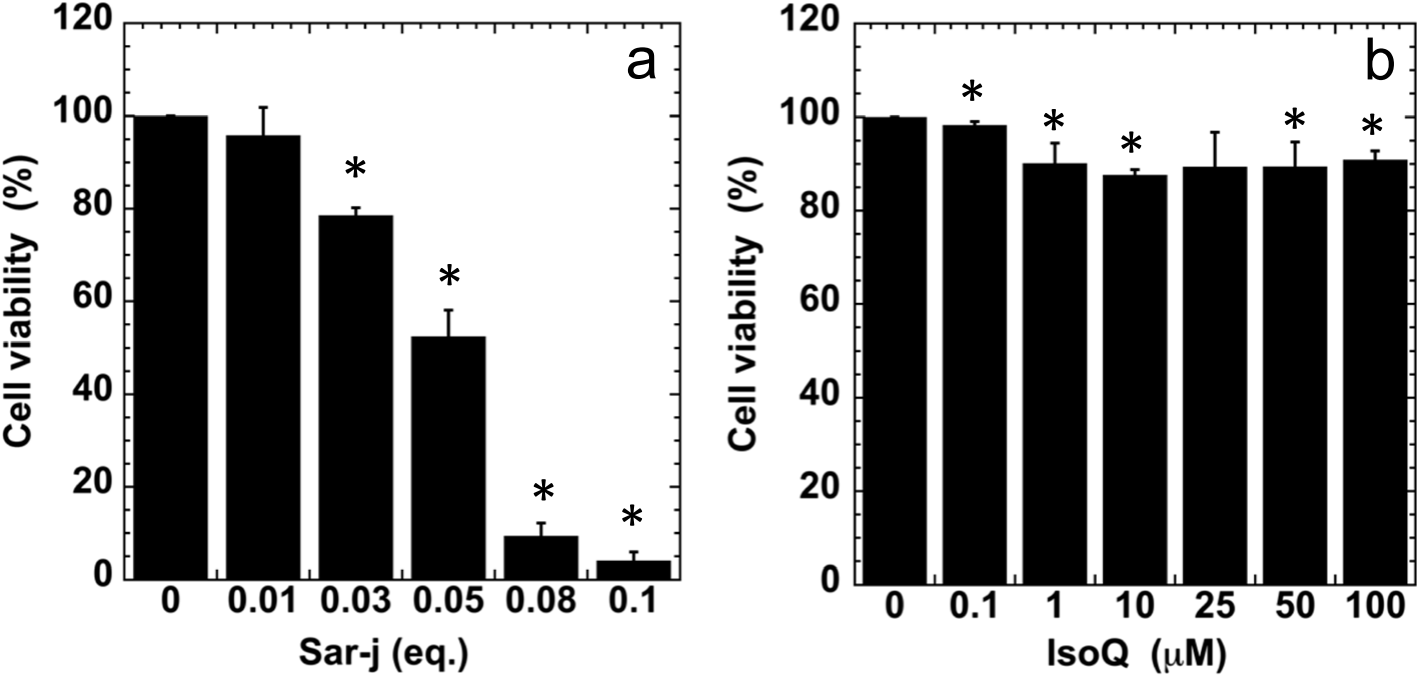
Effect of sar-j (**a**) and isoQ (**b**) on A549 cell viability. Experiment was repeated thrice and SD is indicated with bar (*n* = 5). * Significantly different from each negative control (sar-j = 0 or isoQ = 0) at *P* < 0.05

To determine their cytotoxicity, A549 cells were treated with various concentrations of sar-j and isoQ and cell viability was assessed using the MTT assay. Cell survival was approximately 50% with 0.05 eq. of sar-j (Fig. 4a) and more than 90% between 0.1 mM to 100 mM isoQ (Fig. 4b). Thus, 0.05 eq. of sar-j and 100 μM isoQ was used for further studies.

**Fig. 5:**
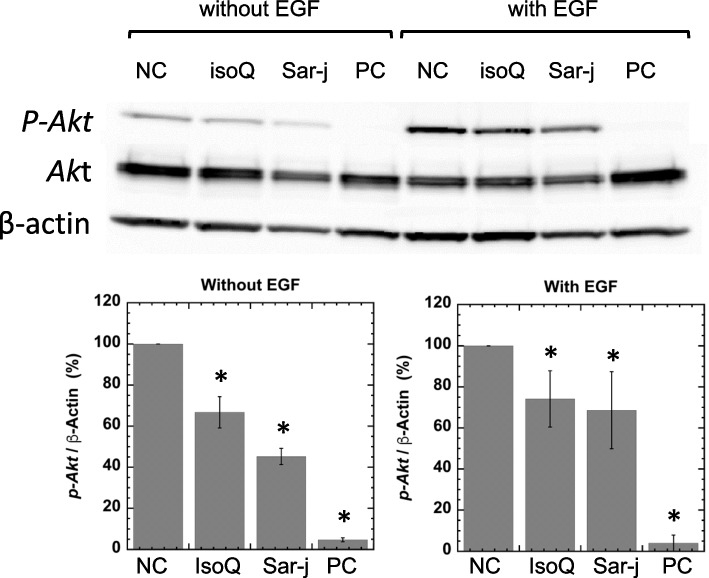
Effects of sar-j (final conc., 0.02 eq. of original juice) and isoQ (50 μM) on Akt phosphorylation (with or without EGF stimulation) in A549 cells. Experiment was repeated thrice and SD is indicated with bar (n = 5). * Significantly different from negative control (NC, sar-j = 0 and isoQ = 0) at *P* < 0.05. Positive control (PC); LY294002 (final 50 μM) served as positive kinase-inhibitor control was added to the PC dish 1 h prior to termination of incubation

We examined the phosphorylation of p-Akt in A549 cells following treatment with sar-j or isoQ. In the absence of EGF stimulation, the levels of p-Akt decreased following treatment with both sar-j and isoQ (Fig. 5, left panel). Following EGF stimulation, the level of p-Akt decreased with sar-j and isoQ treatments (Fig. 5, right panel).

## Discussion

Previously, we revealed the anti-tumorigenic activity of sar-j in skin carcinogenesis in vivo [7]. In this study, we found that NNK-induced lung tumorigenesis in mice was also suppressed following oral intake of sar-j (Table 1, Figs. 1 and 2). We examined several possible anticarcinogenic mechanisms of sar-j, such as antimutagenic activity, protection against DNA damage, and suppression of signal transduction-related cancer proliferation. NNK is metabolically activated to yield methane diazohydroxide and/or methyldiazonium ions, which react with DNA to produce N7meG, O6meG, O^4^-methylthymine, and methyl adducts are consistently detected in the lungs of smokers [14]. We hypothesizes that sar-j inhibit the mutagenicity of the alkylating agents, NNK and MNNG. As expected, the mutagenicity of NNK and MNNG detected using *S. typhimurium* TA1535 decreased in the presence of sar-j (Fig. 3a, c). However, the mutagenicity detected with *S. typhimurium* YG7108 did not decreased with sar-j (Fig. 3a, c). This suggests that the antimutagenic effects of sar-j are likely mediated via *ogt*_*ST*_ and *ada*_*ST*_ enhanced DNA damage repair. Sar-j may enhance O^6^-methylguanine DNA methyltransferases in *S. typhimurium* TA1535 to decrease the mutagenicity detected with *S. typhimurium* TA1535. Therefore, mutagenicity detected with *S. typhimurium* YG7108 (lacking O^6^-methylguanine DNA methyltransferases) was not suppressed by sar-j (Fig. 3a, c).

NNK initially presented as inactive form. Upon exposure to specific enzymes (such as NAD(P) H quinone reductase 1, myeloperoxidase, microsomal epoxide hydrolase, and CYP2A13), NNK undergoes oxidative and reductive metabolism, leading to its metabolites, 4-(methylnitrosaminmo)-1-(3-pyridyl)-1-butanol (NNAL) and NNAL-Glucs [26]. Metabolite detoxification of NNAL-Glucs subsequently leads to the activation of NNK. Previously, we have demonstrated that sar-j inhibited the activity of CYP1A1, CYP1A2, glutathione S-transferase, whereas increased the activity of UDP-glucuronosyltransferase [7]. Sar-j may enhance the metabolically activation of NNK via enhanced glucuronidation, and the NNK-induced mutagenicity in *S. typhimurium* YG7108 increased in the presence of sar-j (Fig. 3a). IsoQ is a water-soluble, heat-labile phenolic compound, which is expected to be an antimutagenic components in sar-j [7]. However, the mutagenicity of NNK and MNNG detected using *S. typhimurium* TA1535 was not inhibited with isoQ (up to 250 nmole), more content than that in sar-j (Fig. 3b, d). This suggests that isoQ may not be the critical component that mediates the antimutagenic effects of sar-j, and there may be other unidentified components in sar-j that enhance the repair of DNA methylation.

Previously, we revealed that oral intake of yamabudo-fr or 2,6-dimethoxy-1,4-benzoquinone (DBQ) provides significant protection against NNK-induced lung tumorigenesis in a mouse model, and that MNNG-induced alkyl-DNA adducts formation in A549 cells was reduced in the presence of yamabudo-fr or DBQ [5]. DNA adducts associated with tobacco smoking may serve as a marker of biologically effective doses of tobacco carcinogens [27]. Sar-j may reduce cellular DNA damage and accelerate the repair of DNA damage caused by alkylating agents. Sar-j inhibited MNNG-induced DNA methylation in A549 cells (supplemental data). Induction of DNA damage by these agents is an important first step in the process of carcinogenesis [28]. Accelerate repair of alkyl-DNA damages is a potential anti-tumorigenic mechanism of sar-j.

We also investigated the effects of sar-j on the later stage of carcinogenesis in A549 cells. Overexpression and mutation of the epidermal growth factor receptor (EGFR) are associated with tumor development [29], and mutant EGFR selectively activates AKT signaling pathways [30]. Mutations or increased expression of members of the ErbB family, including those of the PI3K/Akt pathway, are associated with several malignancies, including lung carcinoma [31]. Phosphorylation of Akt in A549 cells was significantly decreased in the presence of sar-j with or without EGF stimulation, indicating that sar-j components suppress the PI3K/AKT signaling pathways (Fig. 5). IsoQ also suppressed Akt phosphorylation in A549 cells (Fig. 5). Grade of malignancy in the tumor cells (group II) was low when compared to the group I (Fig.1). Although the size of nodules did not change with sar-j or isoQ (Table 1), sar-j or isoQ may suppress the growth of a cancer cell to be a countable nodule. Tumor nodules that were > 1 mm in diameter were counted. The number of nodules that were > 1 mm in diameter was significantly decreased in mouse received sar-j or isoQ (Table 1). Suppression of Akt-mediated growth signaling by sar-j and isoQ may suppress the growth and development of cancer cells to be tumor nodules. This suggests that suppression of Akt-mediated growth signaling may be another mechanism for the inhibition of lung tumorigenesis, and isoQ is an active constituent of sar-j that suppresses cell proliferation. Although extracellular signal-regulated kinase (ERK) phosphorylation in A549 cells was not suppressed by sar-j or isoQ (data not shown), Chen et al. reported that isoQ inhibits ERK phosphorylation and promotes c-Jun N-terminal kinase (JNK) phosphorylation in pancreatic cancer cells [32]. Suppression of the signaling pathway may be involved in its anti-tumor mechanism.

Further purification and identification of active ingredients in sar-j will be performed in future.

## Conclusion

The present study demonstrated that sar-j and isoQ reduced NNK-induced lung tumorigenesis. Sar-j targets both the initiation and growth/progression steps in carcinogenesis, specifically, via anti-mutagenesis, stimulation repair of alkyl-DNA adducts, suppression of Akt-mediated growth signaling. IsoQ might contribute in part to the biological effects of sar-j via suppression of Akt phosphorylation, but it may not be the main active ingredient.

## Supplementary Information

**Additional file 1: Fig. S.** Effect of sar-j (a, b) on MNNG-induced DNA adduct formation. Percentage (%) of O^6^-methylguanine/guanine (O6meG/G) (a) and N^7^-methylguanine/guanine (N7meG/G) (b) adducts formed in the treated DNA. Experiment was repeated thrice and SD is indicated with bar (*n* = 3).

## Data Availability

Not applicable.

## Abbreviations

MTT: 3-(4,5-dimethyl-2-thiazolyl)-2,5-diphenyl-2H-tetrazolium bromide
DMEM: Dulbecco’s modified Eagle’s medium
EGFR: epidermal growth factor receptor
MNNG: 1-methyl-3-nitro-1-nitrosoguanidine
NNK: 4-(methylnitrosamino)-1-(3-pyridyl)-1-butanone
N7meG: N^7^-methylguanine
O6meG: O^6^-methylguanine
PBS: Phosphate buffered saline
SD: Standard deviation
TBST: Tris buffered saline containing 0.1% Tween 20
