# Supplementary material for: Chemopreventive effects and anti-tumorigenic mechanisms of Actinidia arguta, known as sarunashi in Japan toward 4-(methylnitrosamino)-1-(3-pyridyl)-1-butanone (NNK)- induced lung tumorigenesis in a/J mouse
Source: Genes Environ. 2022 Dec 9;44:26. doi: 10.1186/s41021-022-00255-0 (PMC9733242; doi:10.1186/s41021-022-00255-0)
Supplement: Supplementary file 1 — Additional file 1: Fig. S. Effect of sar-j (a, b) on MNNG-induced DNA adduct formation. Percentage (%) of O6-methylguanine/guanine (O6meG/G) (a) and N7-methylguanine/guanine (N7meG/G) (b) adducts formed in the treated DNA. Experiment was repeated thrice and SD is indicated with bar (n = 3). [file 41021_2022_255_MOESM1_ESM.zip › Supplemental data.docx]

Supplemental data

Effects of sar-j on DNA adduct formation in A549 cells treated with MNNG

Materials

*O*^6^-Methylguanine (O6meG) and *N*^7^-methylguanine (N7meG) were purchased from Sigma-Aldrich (Tokyo, Japan).

Methods

Sar-j was defrosted, centrifuged 9000 ×g for 20 min at 20 °C, and supernatant was passed through a 0.45 mM filter for sterilization. Samples were diluted in sterile phosphate-buffered saline (PBS) to obtain concentrations ranging from 0 to 100 mL eq. of the original juice in 10 mL PBS. A549 cells were maintained in Dulbecco's modified Eagle's medium (DMEM) supplemented with 10% heat-inactivated fetal bovine serum and 1% penicillin/streptomycin, and grown in an incubator at 37 °C in a humidified atmosphere of 5% CO_2_. The ability to interfere with methyl adduct formation in DNA was determined as follows [S1]. Briefly, cells were cultured in flasks (25 cm^2^) until they reached 80-90% confluence. The medium was removed, and the cells were washed twice with PBS. An aliquot (10 mL) of different concentrations of the sample (sar-j) dissolved in PBS was added and incubated for 1 h. Then, the sample solution was removed, and the cells were washed twice with PBS. Next, 10 mL (final concentration, 0.2 mM) MNNG dissolved in PBS was added. Following 1 h of incubation at 37°C, the MNNG solution was removed. Cells were harvested by trypsin treatment and the cellular DNA was extracted using a ‘Get pure DNA Kit-Cell, Tissue’ (DOJINDO, Kumamoto, Japan). The DNA was dissolved in 0.1 mL of 0.1 N HCl and incubated at 70°C for 30 min. Immediately after treatment, the mixture was cooled on ice, and a two-fold volume of ice-cold ethanol was added and mixed using a vortex mixer for 5 min. The mixture was cooled at -80°C for 30 min and centrifuged at 17400 ×g for 20 min at 4 °C. The supernatant was collected and evaporated to dryness. The residue was dissolved in water and the fractions were analyzed by LC-MSMS (API4000, AB SCIEX, Framingham, MA, USA) in positive mode ESI using an Inertsil ODS-3 column (1.0 × 150 mm) and 5% methanol as the eluent. LC-MS/MS analysis was performed in positive ion mode with a constant neutral scan to monitor the precursor/product ion transitions m/z 152/134 for guanine and m/z 166/148 for O6meG and N7meG.

Results

As NNK-induced lung tumorigenesis in A/J mice involves methylation of guanine residues, and NNK and MNNG mutagenicity are inhibited by sar-j (Fig. Sa, b), we investigated inhibition of DNA-alkylation as a possible mechanism underlying the anti-tumorigenesis effect of sar-j. As N7meG and O6meG formation in the DNA of NNK-treated A549 cells was below the detection limit of LC-MSMS, we examined the effects on DNA methylation in A549 cells treated with MNNG, a methylating agent. Treatment of A549 cells with 0.2 mM MNNG resulting in the formation of N7meG and O6meG in the DNA. The amounts of N7meG/G and O6meG/G in the cells were 0.107 ± 0.0011 and 0.0213 ± 0.0047, respectively. Following pretreatment with sar-j, the formation of O6meG in A549 cells treated with MNNG was significantly decreased in a sar-j dose-dependent manner (Fig. Sa). ID_50_ of sar-j for O6meG/G formation was 50 μL/10 mL of reaction mixture. However, the formation of N7meG in A549 cells treated with MNNG did not decrease significantly in the presence of sar-j (Fig. Sb).

Discussion

As sar-j inhibited the mutagenicity of the alkylating agent MNNG, we expected that sar-j may suppress methyl-adducts formation in the DNA in MNNG-treated A549 cells. As shown in Fig. 2a, sar-j inhibited MNNG-induced DNA methylation in A549 cells. Sar-j may reduce cellular DNA damage and accelerate the repair of DNA damage caused by alkylating agents.

Reference

S1. Arimoto-Kobayashi S, Kaji K, Sweetman GMA, Hayatsu H. Mutation and formation of methyl- and hydroxyl-guanine adducts in DNA caused by N-nitrosodimethylamine and N-nitrosodiethylamine with UVA irradiation. Carcinogenesis, 1997; 18: 2429-33.

Figure legend

Fig. S. Effect of sar-j (a, b) on MNNG-induced DNA adduct formation. Percentage (%) of O^6^-methylguanine/guanine (O6meG/G) (a) and N^7^-methylguanine/guanine (N7meG/G) (b) adducts formed in the treated DNA. Experiment was repeated thrice and SD is indicated with bar (n=3).
