# Supplementary figures and images for: Chemopreventive effects and anti-tumorigenic mechanisms of Actinidia arguta, known as sarunashi in Japan toward 4-(methylnitrosamino)-1-(3-pyridyl)-1-butanone (NNK)- induced lung tumorigenesis in a/J mouse
Source: Genes Environ. 2022 Dec 9;44:26. doi: 10.1186/s41021-022-00255-0 (PMC9733242; doi:10.1186/s41021-022-00255-0)

## Slide 1
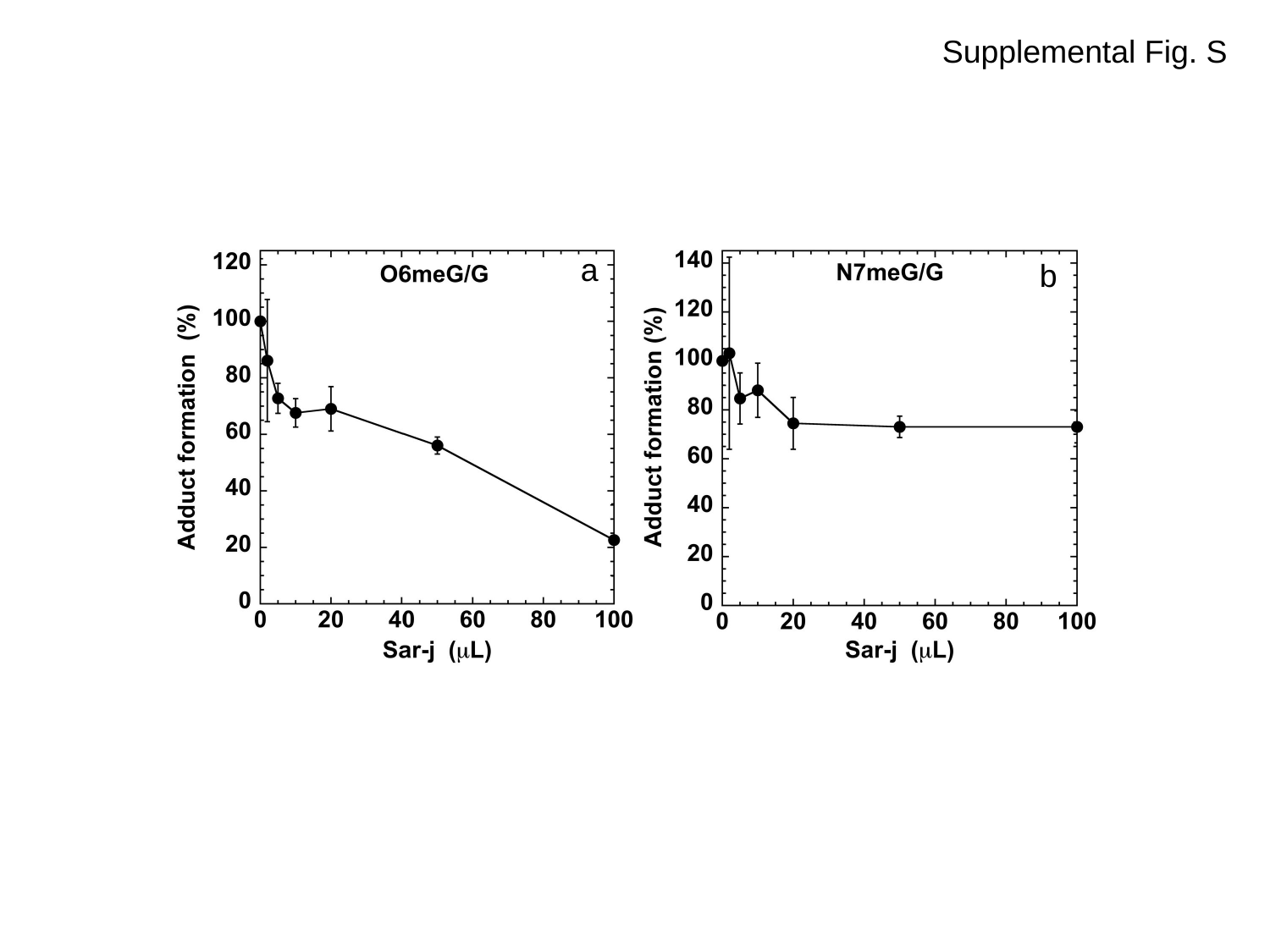

Supplemental Fig. S
a
b

Supplement: Supplementary file 1 — Additional file 1: Fig. S. Effect of sar-j (a, b) on MNNG-induced DNA adduct formation. Percentage (%) of O6-methylguanine/guanine (O6meG/G) (a) and N7-methylguanine/guanine (N7meG/G) (b) adducts formed in the treated DNA. Experiment was repeated thrice and SD is indicated with bar (n = 3). [file 41021_2022_255_MOESM1_ESM.zip › Supplemental Figure S.pptx]
